# ERRATUM: Aberrant DNA hypermethylation of *SDHC*: a novel mechanism of tumor development in Carney triad

**DOI:** 10.1530/ERC-14-0254e

**Published:** 2025-07-18

**Authors:** Florian Haller, Evgeny A Moskalev, Fabio R Faucz, Sarah Barthelmeß, Stefan Wiemann, Matthias Bieg, Guillaume Assie, Jerome Bertherat, Inga-Marie Schaefer, Claudia Otto, Eleanor Rattenberry, Eamonn R Maher, Philipp Ströbel, Martin Werner, J Aidan Carney, Arndt Hartmann, Constantine A Stratakis, Abbas Agaimy

**Affiliations:** ^1^ Institute of Pathology, University Hospital Erlangen, Friedrich-Alexander University Erlangen-Nuremberg, Erlangen, Germany; ^2^ Program on Developmental Endocrinology and Genetics, *Eunice Kennedy Shriver* National Institute of Child Health and Human Development, National Institutes of Health, Bethesda, Maryland, USA; ^3^ Division Molecular Genome Analysis, German Cancer Research Center (DKFZ), Heidelberg, Germany; ^4^ Division of Theoretical Bioinformatics, German Cancer Research Center (DKFZ), Heidelberg, Germany; ^5^ Institut Cochin, INSERM U1016, CNRS UMR 8104, Université Paris Descartes, Sorbonne Paris Cité, Paris, France; ^6^ Department of Endocrinology, Referal Center for Rare Adrenal Diseases, Assistance Publique Hôpitaux de Paris, Hôpital Cochin, Paris, France; ^7^ Institute of Pathology, University Medical Center, Georg-August University, Göttingen, Germany; ^8^ Institute of Pathology, University Hospital, Albert-Ludwigs University Freiburg, Freiburg, Germany; ^9^ School of Clinical and Experimental Medicine, College of Medical and Dental Sciences, Centre for Rare Diseases and Personalised Medicine, Birmingham Women’s Hospital, University of Birmingham and West Midlands Regional Genetics Service, Birmingham, UK; ^10^ Department of Medical Genetics, University of Cambridge, Cambridge, UK; ^11^ Laboratory Medicine and Pathology, Emeritus Staff, Mayo Clinic, Rochester, Minnesota, USA

The journal and publisher apologise for errors in the above paper, which appeared in volume 21 part 4, pages 567–577 . The journal was alerted to the potential manipulation of Fig. 1G given on page 570. After investigation by the authors and journal staff, it was found that the journal typesetter had copied or cloned other areas of the image, in order to generate plausible background under the panel label ‘G’, to relabel the panel. Similar methods must also have been used in panels A, B, C, D, E and F.

The relabelling of Fig. 1 by the journal had no impact on the results, interpretation or conclusions of the paper.

The original file supplied by the authors for Fig. 1 is given in full below:

**Figure 1:**
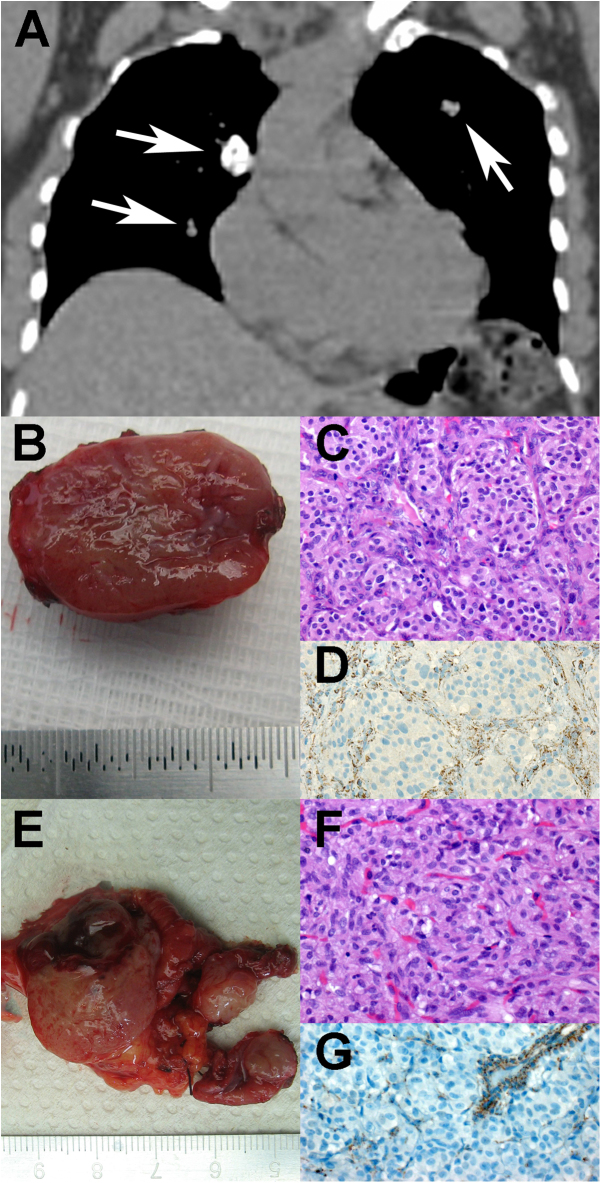
Clinicopathological presentation of a patient with complete form of Carney triad (CT-1). (A) Coronal reconstruction of thoracic CT scan displaying three pulmonary nodules (arrows) consistent with pulmonary chondroma on biopsy. (B) Gross specimen of the resected PGL of the carotid body. (C) Histologic examination reveals the typical ‘Zellballen’ morphology of PGL (H&E, ×400). (D) SDHB immunostaining is absent in the tumor cells but preserved in the surrounding sustentacular cells and endothelial cells (SDHB, ×400). (E) Gross specimen of the gastric GIST. Note the multinodular appearance. (F) Histologic examination displays a characteristic epithelioid morphology (H&E, ×400). (G) SDHB immunostaining is lost in the tumor cells but preserved in the endothelial cells.

